# Genome-Wide Variation in Betacoronaviruses

**DOI:** 10.1128/JVI.00496-21

**Published:** 2021-07-12

**Authors:** Katherine LaTourrette, Natalie M. Holste, Rosalba Rodriguez-Peña, Raquel Arruda Leme, Hernan Garcia-Ruiz

**Affiliations:** a Nebraska Center for Virology, University of Nebraska-Lincoln, Lincoln, Nebraska, USA; b Department of Plant Pathology, University of Nebraska-Lincoln, Lincoln, Nebraska, USA; c Complex Biosystems Interdisciplinary Life Sciences Program, University of Nebraska-Lincoln, Lincoln, Nebraska, USA; Cornell University

**Keywords:** COVID-19, MERS-CoV, S protein, SARS-CoV, SARS-CoV-2, coronavirus, genomic variation, glycoprotein S, protein S, vaccine

## Abstract

The *Severe acute respiratory syndrome coronavirus* (SARS-CoV) and SARS-CoV-2 originated in bats and adapted to infect humans. Several SARS-CoV-2 strains have been identified. Genetic variation is fundamental to virus evolution and, in response to selection pressure, is manifested as the emergence of new strains and species adapted to different hosts or with novel pathogenicity. The combination of variation and selection forms a genetic footprint on the genome, consisting of the preferential accumulation of mutations in particular areas. Properties of betacoronaviruses contributing to variation and the emergence of new strains and species are beginning to be elucidated. To better understand their variation, we profiled the accumulation of mutations in all species in the genus *Betacoronavirus*, including SARS-CoV-2 and two other species that infect humans: SARS-CoV and *Middle East respiratory syndrome coronavirus* (MERS-CoV). Variation profiles identified both genetically stable and variable areas at homologous locations across species within the genus *Betacoronavirus*. The S glycoprotein is the most variable part of the genome and is structurally disordered. Other variable parts include proteins 3 and 7 and ORF8, which participate in replication and suppression of antiviral defense. In contrast, replication proteins in ORF1b are the least variable. Collectively, our results show that variation and structural disorder in the S glycoprotein is a general feature of all members of the genus *Betacoronavirus*, including SARS-CoV-2. These findings highlight the potential for the continual emergence of new species and strains with novel biological properties and indicate that the S glycoprotein has a critical role in host adaptation.

**IMPORTANCE** Natural infection with SARS-CoV-2 and vaccines triggers the formation of antibodies against the S glycoprotein, which are detected by antibody-based diagnostic tests. Our analysis showed that variation in the S glycoprotein is a general feature of all species in the genus *Betacoronavirus*, including three species that infect humans: SARS-CoV, SARS-CoV-2, and MERS-CoV. The variable nature of the S glycoprotein provides an explanation for the emergence of SARS-CoV-2, the differentiation of SARS-CoV-2 into strains, and the probability of SARS-CoV-2 repeated infections in people. Variation of the S glycoprotein also has important implications for the reliability of SARS-CoV-2 antibody-based diagnostic tests and the design and deployment of vaccines and antiviral drugs. These findings indicate that adjustments to vaccine design and deployment and to antibody-based diagnostic tests are necessary to account for S glycoprotein variation.

## INTRODUCTION

Coronaviruses cause respiratory and intestinal infections in animals, including humans. Three species are highly pathogenic to humans: *Severe acute respiratory syndrome coronavirus* (SARS-CoV), first described in China in 2002; *Middle East respiratory syndrome coronavirus* (MERS-CoV), first described in South Arabia in 2012 (1); and *Severe acute respiratory syndrome coronavirus 2* (SARS-CoV-2), first detected in December 2019 in Wuhan, China (2, 3).

Coronaviruses consist of a group of four genera (*Alphacoronavirus*, *Betacoronavirus*, *Gammacoronavirus*, and *Deltacoronavirus*) in the order *Nidovirales*, family *Coronaviridae*, subfamily *Coronavirinae* (2, 3). Gammacoronaviruses and deltacoronaviruses infect birds, and some species infect mammals. Alphacoronaviruses and betacoronaviruses (β-CoVs) infect mammals, primarily bats and humans (1). The genus *Betacoronavirus* contains five subgenera (Table 1): *Embevorirus*, *Merbecovirus*, *Nobecovirus*, *Hibecovirus*, and *Sarbecovirus* (2, 3). The subgenus *Sarbecovirus* contains species that infect bats or humans. Among species that infect humans, the closest relatives to SARS-CoV-2 are SARS-CoV and MERS-CoV (Table 1) (3, 4).

**TABLE 1:** Betacoronavirus (order *Nidovirales*, family *Coronavirida*e, subfamily *Coronavirinae*) nucleotide accessions used in this study^*a*^

Subgenus species	No. of accessions	No. of complete genomes	Reference accession no.	Length (nt)
Embecovirus				
Bovine coronavirus	1148	111	AF220295.1	31,100
Camel coronavirus HKU23	22	9	MN514966.1	31,075
Canine respiratory coronavirus	60	3	LR721664.1	31,190
Equine coronavirus	37	4	EF446615.1	30,992
Human coronavirus HKU1	416	48	DQ415901.1	30,097
Human coronavirus OC43	1386	178	MN306053.1	30,818
Murine coronavirus	258	38	AC_000192.1	31,526
Porcine hemagglutinating encephalomyelitis	92	13	KY994645.1	30,684
Rattus coronavirus HKU24	4	4	NC_026011.1	31,249
Hibecovirus				
Bat Hp-betacoronavirus Zhejiang2013	1	1	NC_025217.1	31,491
Merbecovirus				
Betacoronavirus erinaceus	10	6	KC545386.1	30,175
Bat coronavirus HKU4	87	11	EF065508.1	30,316
Bat coronavirus HKU5	66	10	MH002342.1	30,529
MERS-CoV	1351	572	MG987420.1	30,484
Nobecovirus				
Rousettus bat coronavirus HKU9-1	116	10	EF065516.1	29,155
Rousettus bat coronavirus GCCDC1	27	3	NC_030886.1	30,161
Sarbecovirus				
Bat coronavirus	2	2	GU190215	29,276
Bat SARS coronavirus	45	28	MN996532.1	29,855
Bat SARS-like coronavirus	147	19	KY417150.1	30,311
SARS-CoV	637	254	MK062183.1	29,874
SARS-CoV-2	2,379	2,315	NC_045512.2	29,903

a For each virus species, one annotated accession describing the full genome was used as a reference. Only accessions describing complete genomes were used for nucleotide variation analyses. Accessions were downloaded from NCBI on 6 April 2020. For SARS-CoV-2, additional accessions were downloaded on 13 May 2020.

β-CoVs have a monopartite, linear, positive-strand RNA genome of approximately 30,000 nucleotides (nt). The virion is spherical, enveloped, and about 120 nm in diameter (1, 5). Genomic RNA associates with the nucleoprotein (N) to form a nucleocapsid. The membrane (M) protein forms part of the envelope, which also contains the small membrane E protein. The virion surface displays spikes formed by the S glycoprotein (6–8) that mediate cell entry by interacting with cellular receptors and entry cofactors (9–12). The S glycoprotein is divided into S1 and S2 subunits that are separated by proteolytic cleavage via cellular proteases and cofactors (12–15). The virion contains large and small spikes formed by S1 and S2 together and by S2 subunits, respectively (8). In subunit S1, the carboxyl-terminal domain contains a core and the receptor binding subdomains (1, 7–11).

Several lines of evidence show that β-CoVs, including SARS-CoV-2, are evolving and accumulate mutations in their genome (1, 16). For RNA viruses, important sources of genetic variation are RNA recombination and nucleotide insertions, deletions, and substitutions introduced during RNA replication (17). Genetic variation combined with selection pressure imposed by genetically diverse hosts favors the accumulation of mutations that support the emergence of new virus strains and species (18–20). These general principles of virus evolution explain several features of β-CoVs, including SARS-CoV-2. Both SARS-CoV and SARS-CoV-2 likely emerged through RNA recombination of two species infecting bats. The recombinant progeny then adapted to infect humans (3). Within a year of the initial description, several SARS-CoV-2 strains have been detected that mainly accumulate mutations in the S glycoprotein (16, 21). Temporal and spatial genetic relationships show accumulation of mutations in the genome, allowing SARS-CoV-2 isolates to be differentiated (22, 23). In the United States, a variant carrying the Q677P substitution in the S glycoprotein is now abundant in the southwest, and new mutations within this variant have created sublineages (24). Furthermore, variants infecting the same individual have been detected (20, 25–27).

The S glycoprotein is the common target for neutralizing antibodies developed in response to natural infection or vaccines (28–31). Neutralizing antibodies are formed against the prefusion conformation of the entire S glycoprotein. In contrast, nonneutralizing antibodies are formed against the S2 subunit (8, 32). Antibodies against the S glycoprotein are used as markers in diagnostic assays (28, 29, 32). Accordingly, the emergence of new variants with mutations in the S glycoprotein has the potential to compromise the efficacy of vaccines and the immunity mediated by natural infection (20, 27, 33–36). Conversely, antibodies developed through natural infection or vaccines may impose selection pressure on β-CoVs (20, 36). For these and other reasons, it is imperative to understand the biological properties of β-CoVs that contribute to the emergence of new strains and species.

Virus variation, evolution, and adaptation to diverse hosts are mediated by genetic determinants in the viral genome and selection pressure imposed by the host (37–40). Accordingly, characterization of genomic variation is fundamental to our understanding of β-CoV evolution and host adaptation. We hypothesized that genetic determinants of variation in β-CoVs are conserved across species, including SARS-CoV-2. In this study, we profiled the genomic variation in all species in the genus *Betacoronavirus*. Genome-wide nucleotide variation analyses combined with amino acid variation analyses revealed that variation patterns are conserved across β-CoVs, including the presence of variable areas at homologous locations. The most variable parts are the S glycoprotein, followed by open reading frame 8, accessory proteins 3 and 7, and the N protein. Genome-wide distribution of mutations of all β-CoVs provides an explanation for the emergence of new β-CoV species, such as SARS-CoV-2, and for the emergence of strains. These findings and published results (16, 20, 24, 41, 42) predict how and where SARS-CoV-2 will accumulate mutations and differentiate into new biological strains. SARS-CoV-2 will likely evolve as it adapts to genetically diverse human populations (20, 43) and possibly to selection constraints imposed by vaccines, antiviral drugs, and antibodies developed against natural infections (20, 30, 36). Our results underscore the potential for the continual emergence of new β-CoV species and strains with novel biological properties.

## RESULTS

### S glycoprotein is more variable than the rest of the genome.

**FIG 1:**
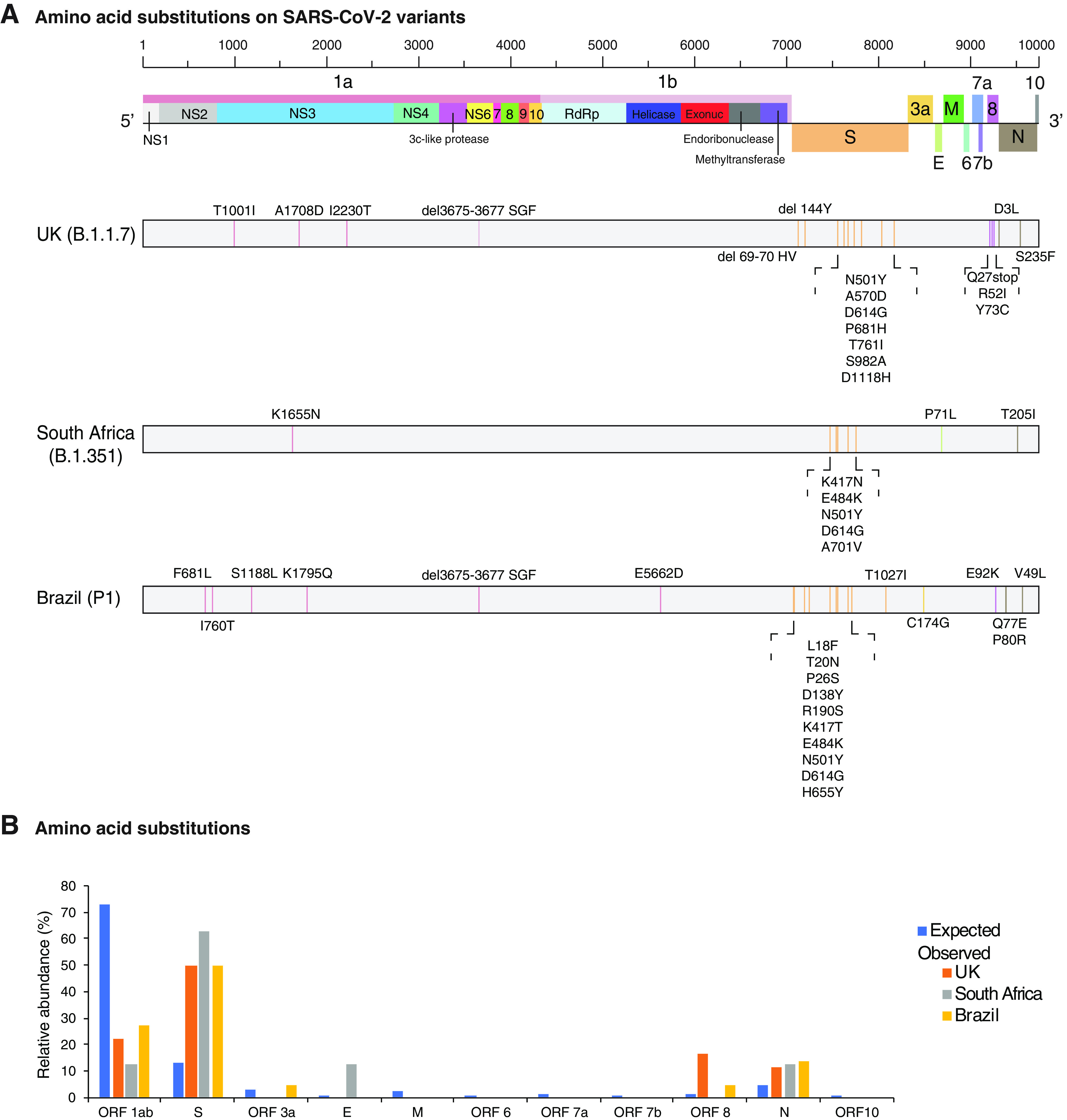
Amino acid substitutions in UK, South Africa, and Brazil SARS-CoV-2 strains. (A) Location of amino acid mutations on the SARS-CoV-2 genome. Coordinates are based on accession no. NC_045512.2. (B) Proportion of amino acid substitutions observed and randomly expected based on the length of each protein or open reading frame. Strains are color coded.

SARS-CoV-2 strains identified to date (16, 21) differ in the accumulation of nonsynonymous substitutions in the entire genome (Fig. 1A). However, nonsynonymous substitutions preferentially accumulate in the S glycoprotein and in the N protein. In all other parts of the genome, mutations accumulate to a frequency that is equal to or less than that expected randomly (Fig. 1B). New mutations continue to arise and diversify SARS-CoV-2 into variants (20, 24). However, recurrent mutations mapped along the SARS-CoV-2 genome preferentially accumulate in the S glycoprotein (22, 23).

**FIG 2:**
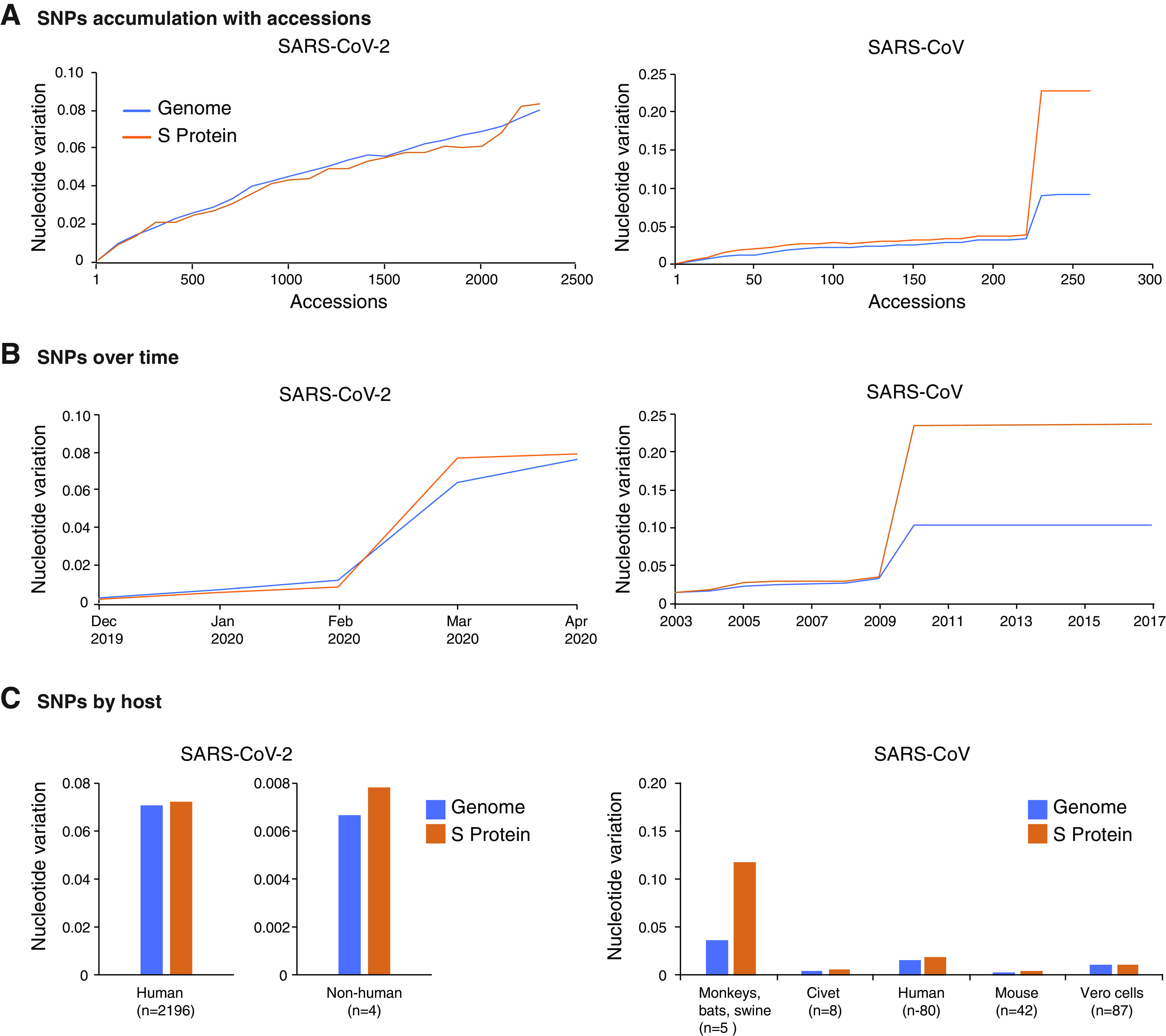
Nucleotide variation in SARS-CoV-2 and SARS-CoV. (A) Nucleotide variation over accessions. (B) Nucleotide variation over time. (C) Nucleotide variation by host. The number of accessions in the analysis is indicated in parenthesis.

To further test preferential accumulation of mutations in the S glycoprotein, we measured single-nucleotide polymorphisms (SNPs) in the entire SARS-CoV-2 genome and separately in the S glycoprotein, normalized to their respective length. SARS-CoV was included in the analysis as it was closely related to SARS-CoV-2. Nucleotide accessions were added in increments of 50 or 10 for SARS-CoV-2 and SARS-CoV, respectively. In both species, the S glycoprotein accumulated more polymorphic sites than the rest of the genome proportionately (Fig. 2A). Using a chronological approach, SARS-CoV-2 accessions were analyzed by month from December 2019 to April 2020. For SARS-CoV, accessions were analyzed by year from 2003 to 2017. The number of polymorphic sites in the S glycoprotein was similar to or higher than that of the rest of the genome in SARS-CoV and SARS-CoV-2 over time (Fig. 2B) and in all hosts (civets, humans, mice, and Vero cells) (Fig. 2C). In SARS-CoV-2, the S glycoprotein represents approximately 17% of the genome (Fig. 1B). However, it accumulated at least 50% of the mutations (Fig. 1B). Accordingly, the S glycoprotein accumulated mutations at a frequency that is at least 3-fold higher than would be expected randomly. The frequency was even higher in accessions derived from nonhuman hosts and in SARS-CoV (Fig. 2C). These results show that the SARS-CoV-2 S glycoprotein is the most variable part of the genome.

### Differentiation of SARS-CoV-2 based on the S glycoprotein.

**FIG 3:**
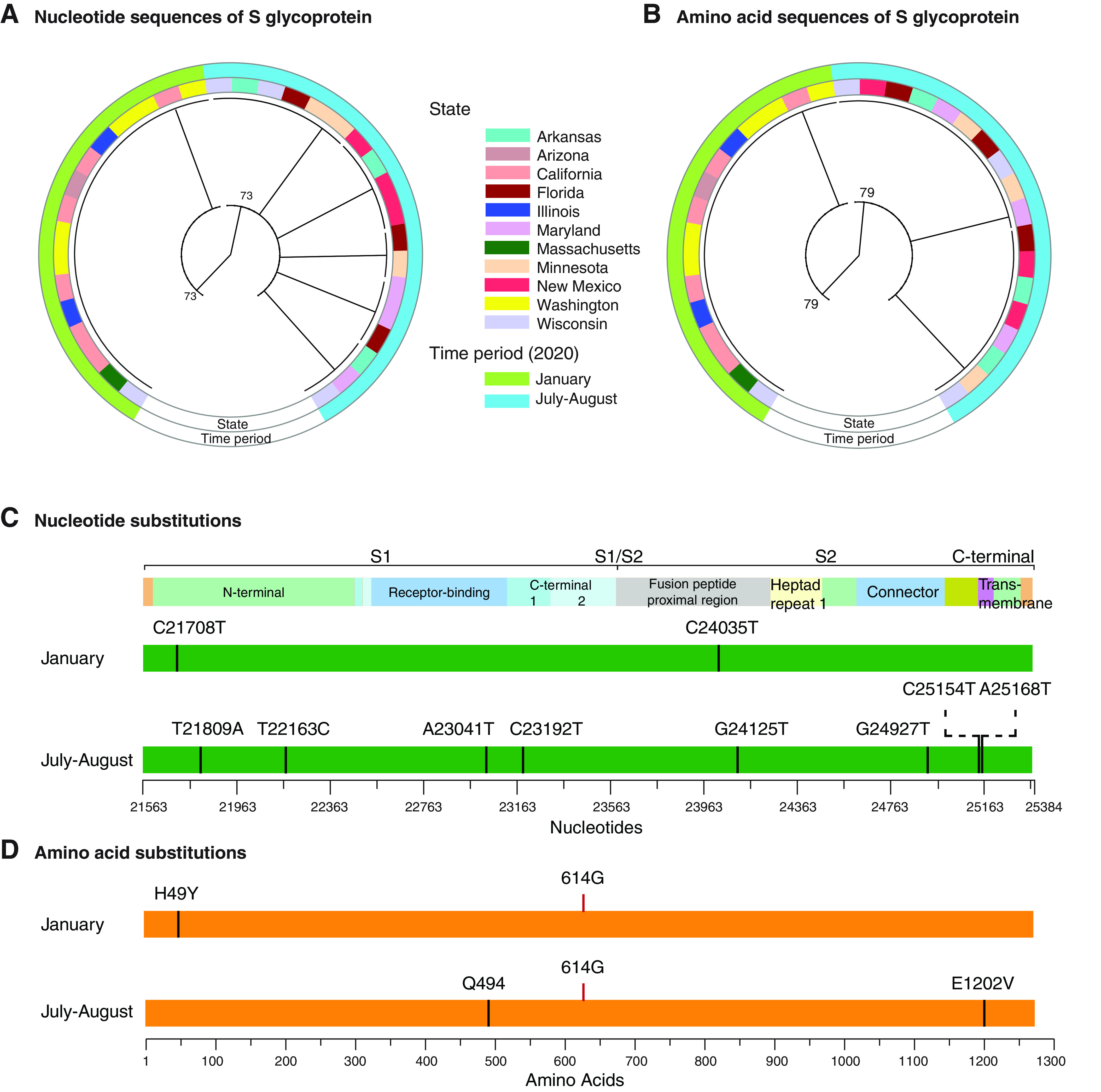
Phylogram and mutations of U.S. SARS-CoV-2 accessions based on the S glycoprotein. (A) Phylogenetic tree of the coding sequence for S protein based on nucleotide accessions from early (January) and middle (July and August) 2020. Color-coded rings indicate the time period and state of origin. (B) Phylogenetic tree of S protein generated using amino acid sequences from early and late time periods. (C) Mutations found in nucleotide accessions from early and late time periods. (D) Mutations found in protein accessions from early and late time periods. Amino acid 614 (G) is indicated.

The variable nature of the S glycoprotein suggests it is a major contributor to SARS-CoV-2 evolution and diversification. To further test this model, using accessions from the United States, we generated a phylogenetic tree using the S glycoprotein and two time periods. Based on nucleotide (Fig. 3A) or amino acid sequence (Fig. 3B), accessions from January 2020 clustered separately from accessions from July and August 2020. With respect to the reference sequence Wuhan-Hu-1 (NC_045512.2), U.S. accessions from January accumulated two nucleotide substitutions (Fig. 3C) and one amino acid (H49Y) substitution that maps to the N-terminal domain of the S1 subunit (Fig. 3D). These mutations were not present on accessions from July and August 2020. Instead, eight new nucleotide (Fig. 3C) and two new amino acid mutations were detected, a Q494L substitution in the receptor binding motif and an E1202V substitution in the transmembrane motif (Fig. 3D). In both time periods, all accessions contained the D614G mutation (Fig. 3D). Consistent with recent observations (16, 24), these results support the model that mutations in the S glycoprotein mediate the emergence of new strains and that the S glycoprotein is a major contributor to SARS-CoV-2 evolution.

### Genome-wide variation in SARS-CoV-2.

**FIG 4:**
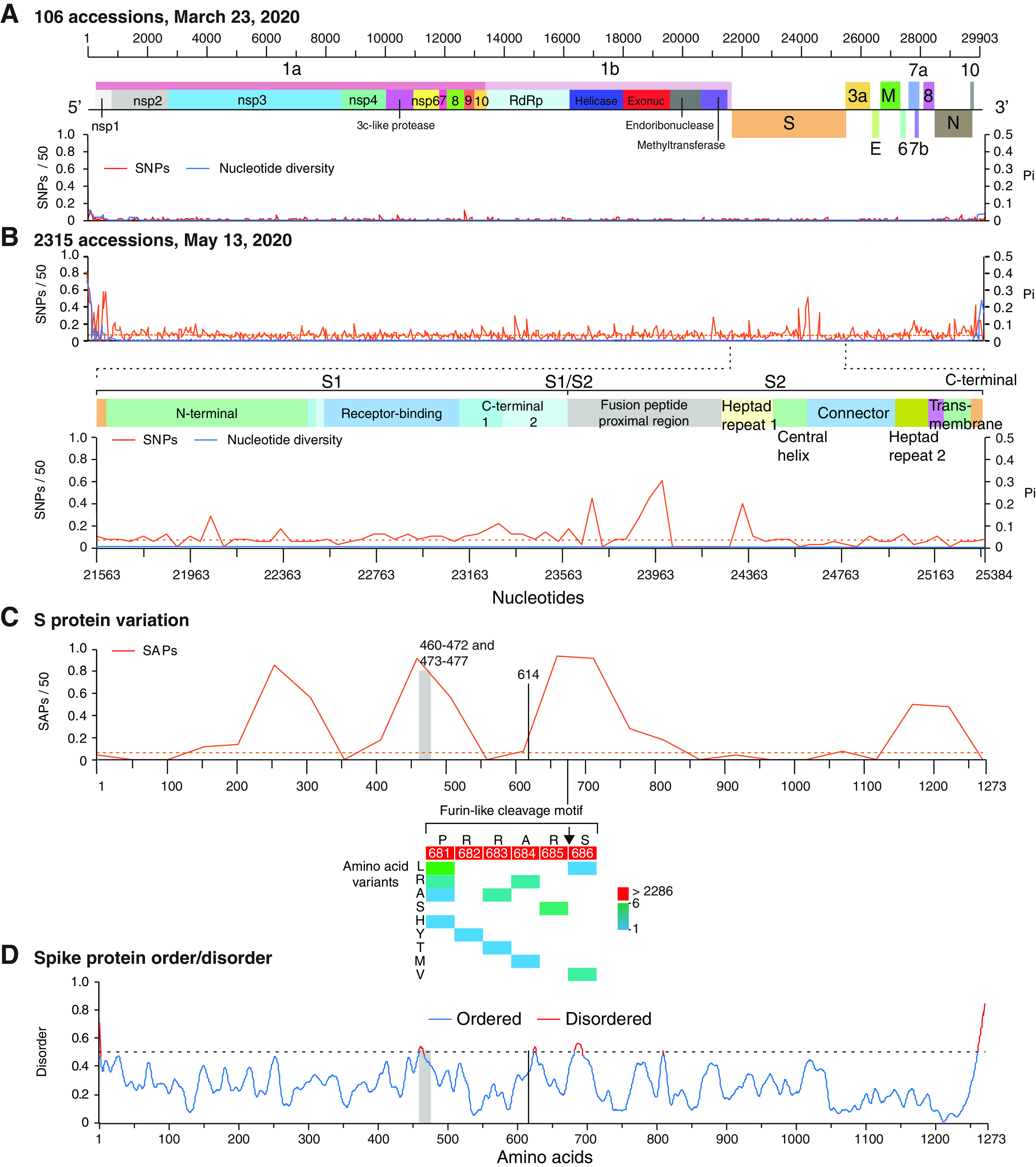
Variation in SARS-CoV-2 genome and S protein. (A) Single-nucleotide polymorphism (SNP) and nucleotide diversity (Pi) plotted with respect to the SARS-CoV-2 genome based on accessions available on 23 March 2020. A 99% confidence interval (*P* < 0.01) is indicated as a horizontal line for each parameter. Nucleotide and amino acid coordinates are based on accession no. NC_045512.2. (B) Variation in SARS-CoV-2 downloaded on 13 May 2020 with 2,315 accessions. (C) Single-amino-acid polymorphism (SAP) plotted with respect to SARS-CoV-2 S protein based on accessions available 14 May 2020. A vertical gray bar represents the ACE2 binding domain. Amino acid 614 is indicated. Mutations in the Furin-like cleavage motif and their frequency are indicated with a heat map. (D) Order and disorder in S protein.

Although nonsynonymous substitutions preferentially accumulate in the S glycoprotein, other areas of the genome also accumulate mutations (Fig. 1). To characterize SARS-CoV-2 genome variation, we compared accessions early and later in the pandemic. Using a 50-nt window, variation in SARS-CoV-2 was measured for the 106 nucleotide accessions available on NCBI on 23 March 2020, the 2,315 nucleotide accessions available on 13 May 2020, and the 2,299 amino acid accessions available on 14 May 2020. No variable areas in the genome were detected in accessions representing the early part of the pandemic (Fig. 4A). However, by May 2020, variation was detected in several areas (Fig. 4B). SNPs were higher than the average of the genome in the S glycoprotein, ORF8, and at the N-terminal part of ORF 1a (Fig. 4B). A single-amino-acid polymorphism (SAP) analysis showed variation in the S glycoprotein maps to the N-terminal domain, the receptor binding domain, the fusion peptide-proximal region, the heptad repeat 2, and the transmembrane domain (Fig. 4C). These results are consistent with the detection of recurrent deletions that map to the N-terminal domain in immunocompromised patients (20). An order/disorder analysis showed that, in SARS-CoV-2, the S glycoprotein has intrinsically disordered areas in the receptor binding domain, C-terminal domain 2, and the fusion peptide proximal region (Fig. 4D). Intrinsically disordered regions often interact with multiple molecular partners, are highly plastic, and show high evolutionary rates (44). Consistent with these results, major mutations (D614G and Q677P) that render the virus more transmissible and pathogenic to humans (24, 34, 45) map near the hypervariable region in the disordered C-terminal domain 2 in S1 (Fig. 4C). The S1/S2 cleavage site is located within a variable region (Fig. 4D). In the U.S. accession subset analyzed here (Fig. 3), no variation was detected in the S1/S2 cleavage site. However, in the larger data set, mutations were detected at the S1/S2 cleavage site (Fig. 4C). Because variation in the S1/S2 cleavage site contributes to cellular tropisms and pathogenesis (13), these results suggest that the S1/S2 cleavage site tolerates mutations and can contribute to SARS-CoV-2 diversification.

Collectively, these observations support the model that the S glycoprotein is variable and mutationally robust and contains intrinsically disordered areas.

### Genome-wide variation in betacoronaviruses.

The variation pattern described above could be a property exclusive to SARS-CoV-2, to a subset of species, or a general property of β-CoVs. To distinguish the difference, we profiled the genome variation in all members of the genus *Betacoronavirus*. To ensure statistical power (46), the analyses described here were based on species with three or more accessions. At least three accessions describing complete genomes were available for 19 of the 21 species in the genus *Betacoronavirus*. The length of the genomes ranged from 29,855 to 31,190 nt (Table 1).

**FIG 5:**
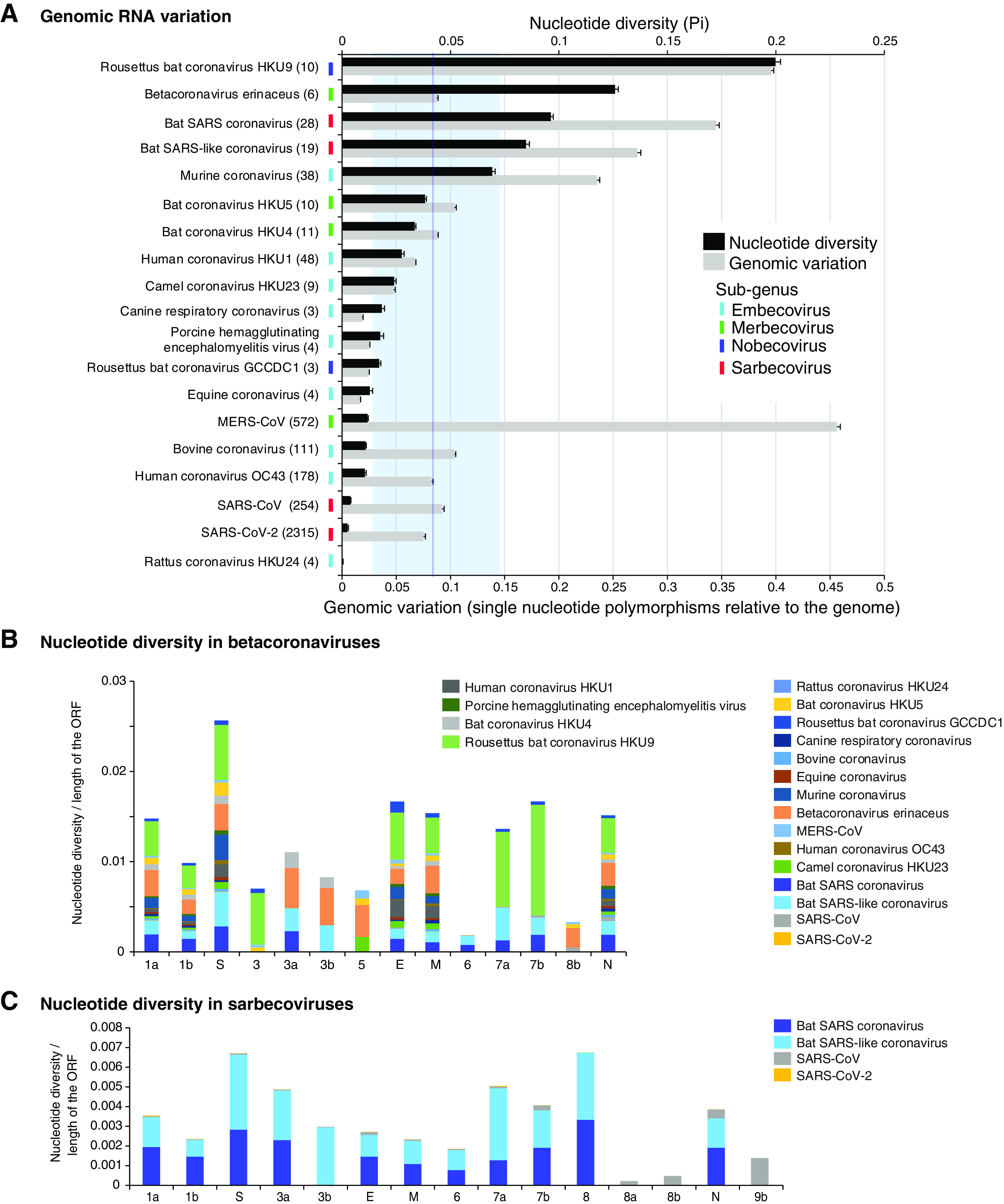
Genomic variation in β-CoVs. (A) Nucleotide variation as determined by nucleotide diversity (Pi) and genomic variation index (proportion of single-nucleotide polymorphisms with respect to the length of the genome). Bars represent the averages and standard errors for each species. The vertical line represents the mean Pi and 99% confidence interval (*P* < 0.01). Virus names are color-coded by genera. (B) Cumulative nucleotide diversity by ORF in the genome of all β-CoVs. (C) Cumulative nucleotide diversity by ORF in the genome of all sarbecoviruses.

We measured nucleotide variation in all members of the genus *Betacoronavirus* (Table 1). SNPs and nucleotide diversity, estimated in a 50-nt window, showed that β-CoVs in general and species in the subgenus *Sarbecovirus* in particular are highly variable (Fig. 5A). Due to its high variability (47), and as a point of comparison, we estimated HIV-1 variation using the same method. The most diversity was observed in *Rousettus bat coronavirus* HKU9, other species infecting bats, and MERS-CoV (Fig. 5A). In these species, more than 25% of the nucleotides in the genome were polymorphic. Genomic variation is not a function of the number of accessions, because similar results were observed using the nucleotide diversity index (Pi), which normalizes for the number of accessions (48) (Fig. 5A).

**FIG 6:**
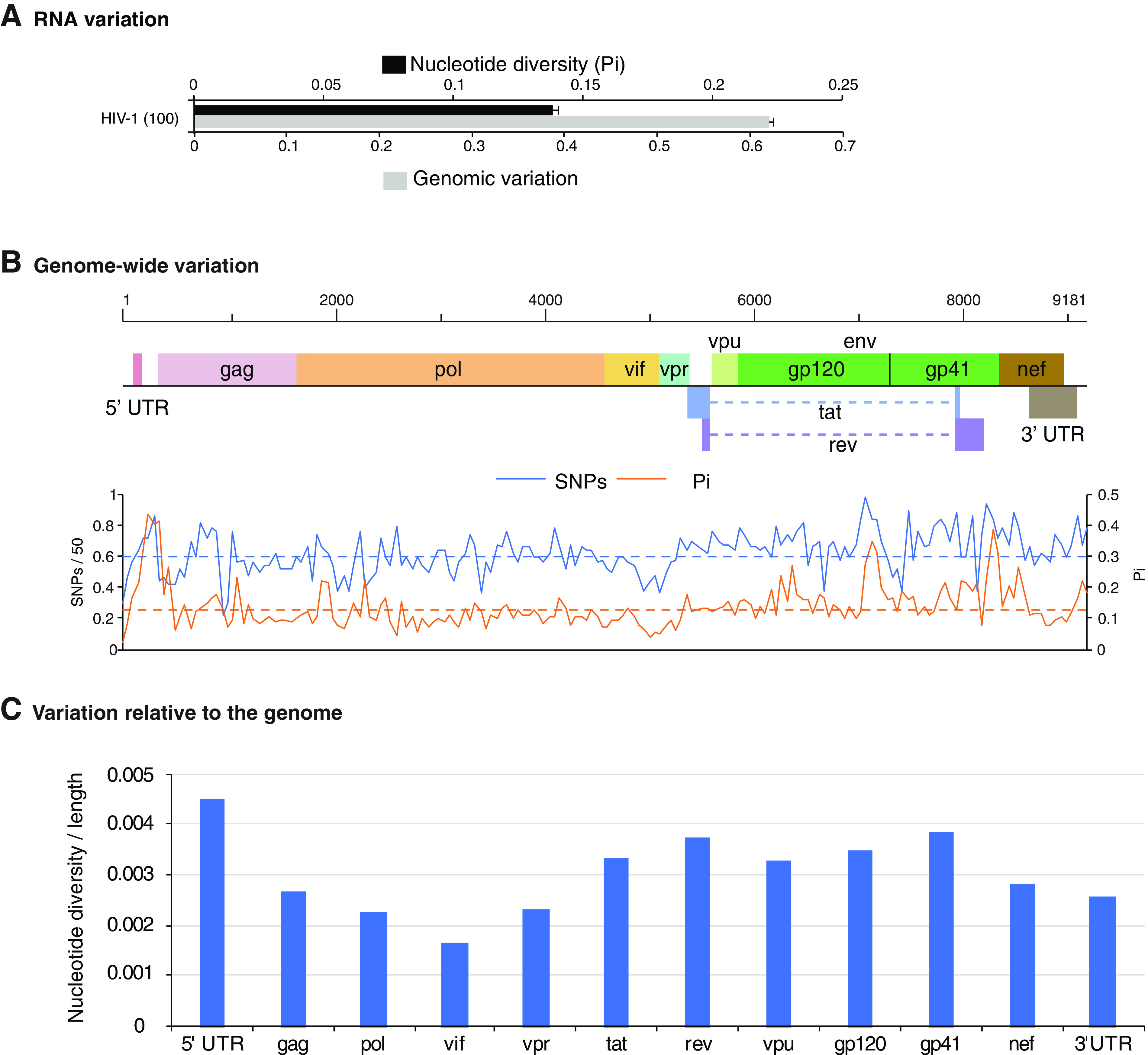
Genomic variation in HIV-1. (A) Nucleotide diversity and genomic variation index (single-nucleotide polymorphisms relative to the genome) estimated using 100 accessions. Bars represent the averages and standard errors. (B) Genome-wide nucleotide variation. Single-nucleotide polymorphism (SNP) and nucleotide diversity (Pi) are plotted with respect to the genome. The average and 99% confidence interval (*P* < 0.01) are indicated as a horizontal line for each parameter. Coordinates are based on accession no. NC_001802.1. (C) Nucleotide diversity normalized to the length of the ORF or UTR.

Variation in HIV-1 (Fig. 6) was higher than that for all β-CoVs, with one exception (Fig. 5A). Based on nucleotide diversity (Pi), the genome of *Rousettus bat coronavirus* accumulated more variation than HIV-1. The next three most variable species included bat SARS coronaviruses, and their nucleotide variation varied from 57% to 86% of that observed for HIV-1 (Fig. 5A). In contrast, nucleotide diversity in SARS-CoV and SARS-CoV-2 was approximately 10% of that observed for HIV (Fig. 5A). Variation estimated for all β-CoVs was higher than that of polioviruses (0.1%), known for being genetically stable (49). The wide range of genomic variation across β-CoV species may reflect a bias in the source of the accessions, such as being obtained from genetically diverse hosts. However, these results show that β-CoVs have the potential to be highly variable.

The genome of β-CoVs consists of 11 to 14 open reading frames (ORFs). Coding regions for accessory and structural proteins located 3′ of the S proteins are not in synteny (1, 50). After normalizing to their length, the most variable parts of the genome were the S glycoprotein, followed by the E protein, protein 7, the M protein, and the N protein (Fig. 5B). The lowest variation was detected in open reading frame 1b (Fig. 5B), which codes for nonstructural proteins that mediate virus replication: RNA-dependent RNA polymerase, RNA helicase, exonuclease, endoribonuclease, and methyltransferase. Within the subgenus *Sarbecovirus*, the most variable part of the genome was the S glycoprotein, followed by ORF8, accessory proteins 3a, 7a, and 7b, and N protein. The lowest variation was detected in open reading frame 1b (Fig. 5C).

**FIG 7:**
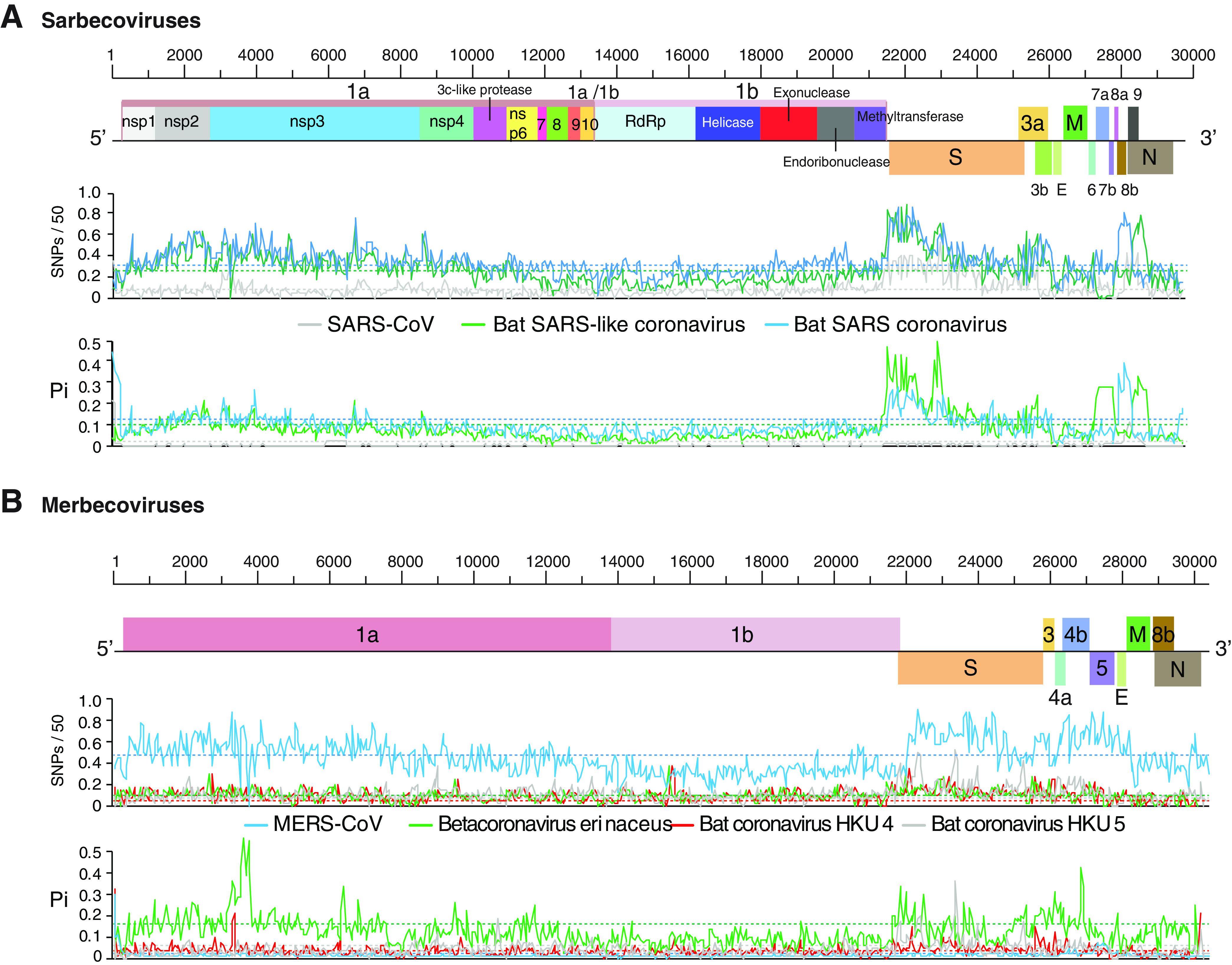
Genome-wide nucleotide variation in sarbecoviruses and merbecoviruses. Single-nucleotide polymorphism (SNP) and nucleotide diversity (Pi) were plotted with respect to the genome. The average and 99% confidence interval (*P* < 0.01) are indicated as a horizontal line for each parameter. Species are represented by colored horizontal lines, and ORFs are color coordinated. (A) Sarbecovirus variation. Coordinates are based on SARS-CoV accession no. MK062183.1. (B) Merbecovirus variation. Coordinates are based on MERS-CoV accession no. MG987420.1.

Genome-wide maps illustrate the distribution of nucleotide variation (Fig. 7). In the S glycoprotein, ORF8, and 3a, nucleotide polymorphisms were higher than the average of the genome. In the two species infecting bats, variation was also detected in nsp1 (inhibits host gene expression) (51), nsp2 (inhibits cell signaling) (52), and nsp3 (papain-like protease) (53). The lowest variation was detected in the 3′ half of ORF1a and in ORF1b (Fig. 7A). This pattern was observed in all species in the subgenus *Sarbecovirus*, including SARS-CoV (Fig. 7A).

**FIG 8:**
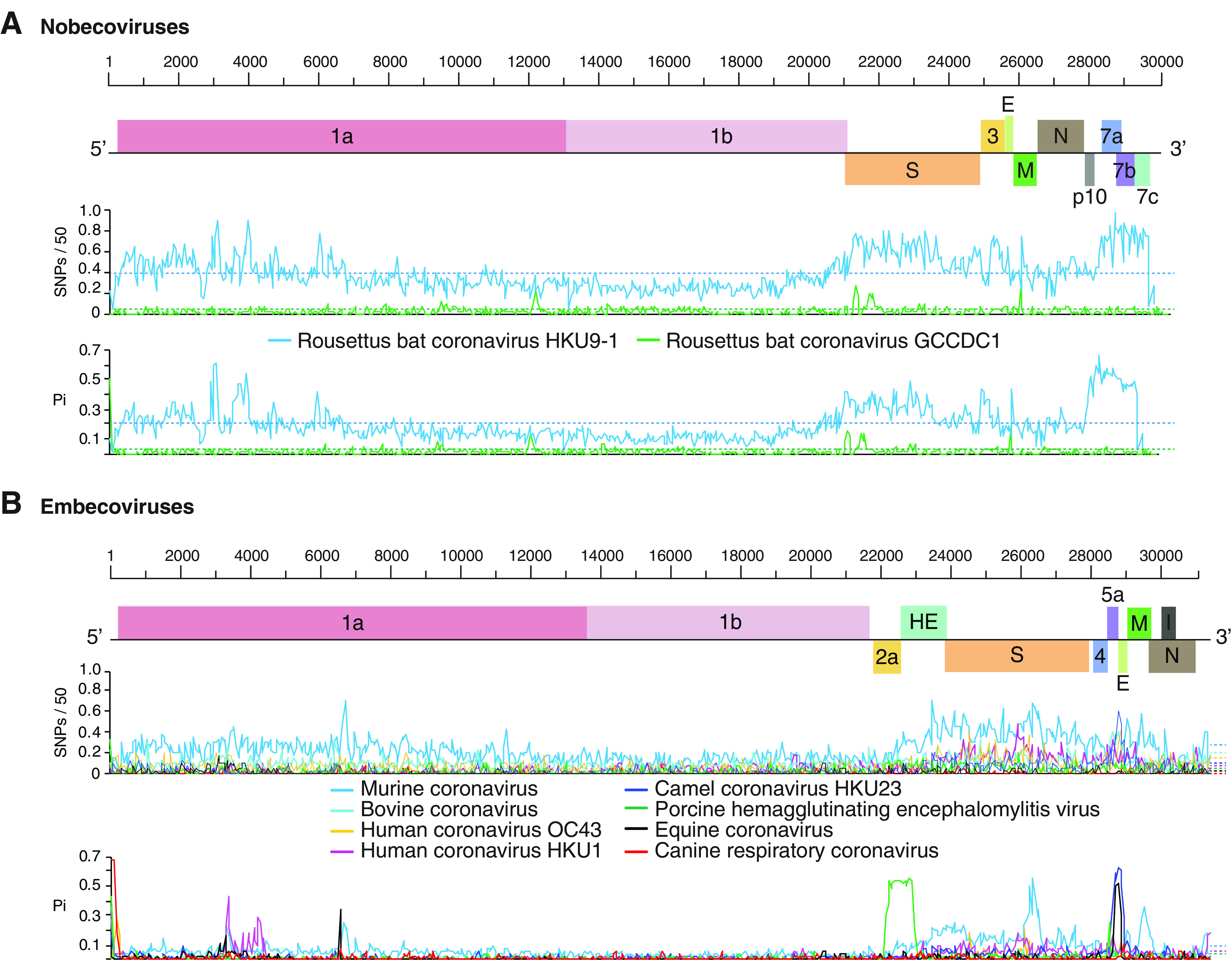
Genome-wide nucleotide variation in nobecoviruses and embecoviruses. Labels are as described for Fig. 7. (A) Nobecovirus variation. Coordinates are based on Rousettus bat coronavirus HKU9 accession no. EF065516.1. (B) Embecovirus variation. Coordinates are based on Bovine coronavirus accession no. AF220295.1.

In the subgenera *Merbecoviruses* (Fig. 7B), *Nobecovirus* (Fig. 8A), and *Embecovirus* (Fig. 8B), the S glycoprotein is the most variable part of the genome. Other areas of hypervariation include nsp1, nsp2, and the nsp3 protease, and the lowest variation was detected in the 3′ half of ORF1a and in ORF1b (Fig. 8).

Collectively, genome-wide variation described above show that, in all members of the genus *Betacoronavirus*, the S glycoprotein is the most variable part of the genome, and replication proteins in ORF1b are the least variable (Fig. 5, 7, and 8).

### Betacoronavirus differentiation into strains.

**FIG 9:**
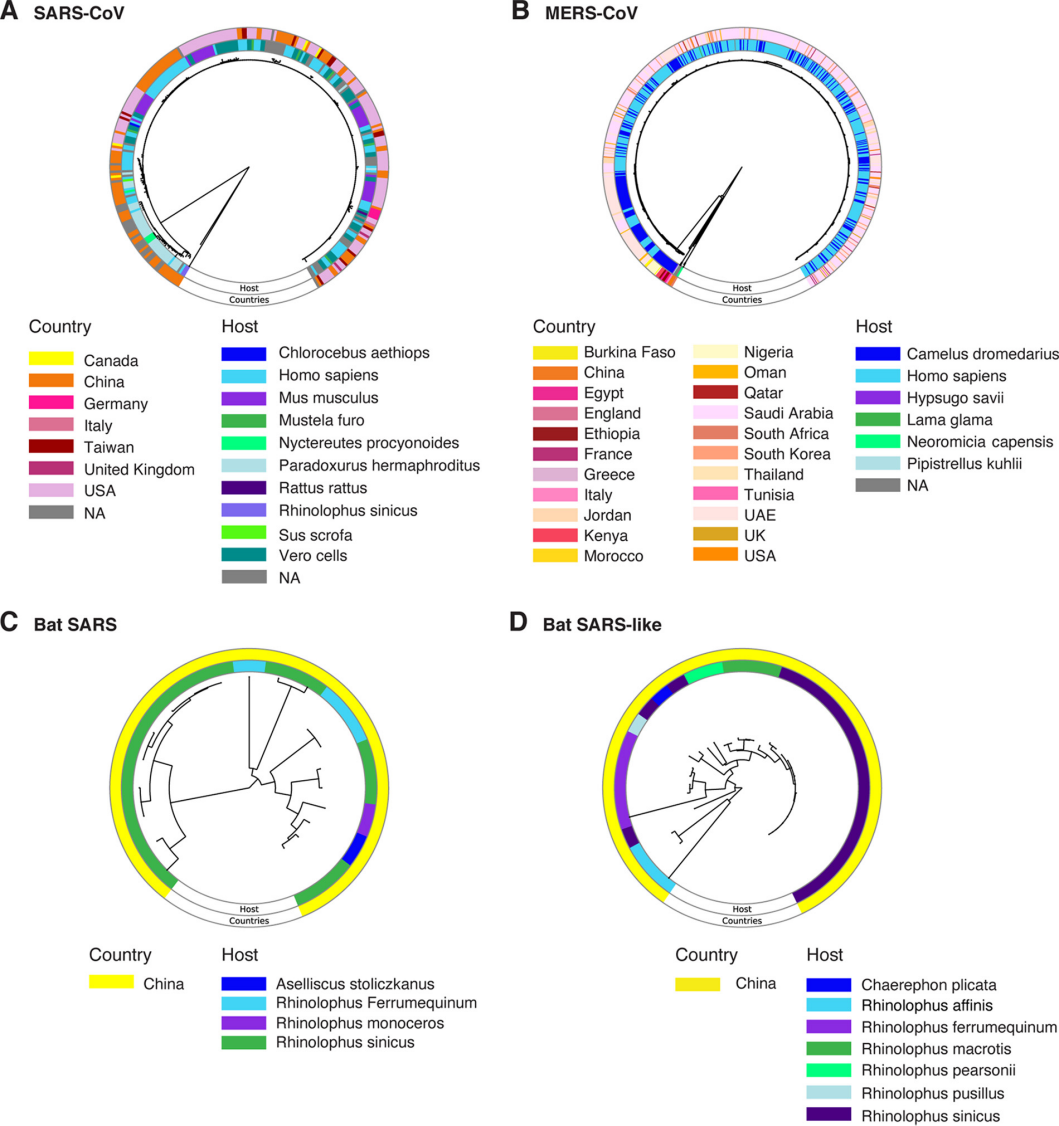
Phylogram based on the coding region for S protein in species closely related to SARS-CoV-2. Color-coded rings indicate hosts and country of origin. (A) SARS-CoV phylogenetic tree based on S protein amino acid sequences. (B) MERS-CoV. (C) Bat SARS coronavirus. (D) Bat SARS-like coronavirus.

The S glycoprotein mediates receptor recognition and membrane fusion during viral entry into the cells (8–11). Consistent with this role, ACE2 receptor binding is a determinant of host range in sarbecoviruses (11, 54–57). For viruses in the subfamily *Torovirinae* within the family *Coronaviridae*, receptor binding is a determinant of host range (58). In the β-CoV genome, as described above, the S glycoprotein is the most variable (Fig. 5B). These observations predict that coronaviruses differentiate into strains based on selection pressure from the host. To test this hypothesis, we generated a phylogeny based on the S glycoprotein for SARS-CoV, MERS-CoV, and closely related species infecting bats. In SARS-CoV (Fig. 9A) and MERS-CoV (Fig. 9B), accessions formed clusters that correlated with the country of origin and host species. Accessions representing bat SARS coronavirus (Fig. 9C) and bat SARS-like coronavirus (Fig. 9D) originated exclusively from China, and accessions clustered according to the host.

These results support the model that, in β-CoVs, variation in the S glycoprotein separates isolates into strains and may reflect the effect of selection imposed by the host. Consistent with this model, reinfection in humans has been confirmed (25, 27). In Brazil, a case of reinfection occurred with a new strain containing an E484K mutation in the S protein (27).

### Amino acid variation in the S glycoprotein.

**FIG 10:**
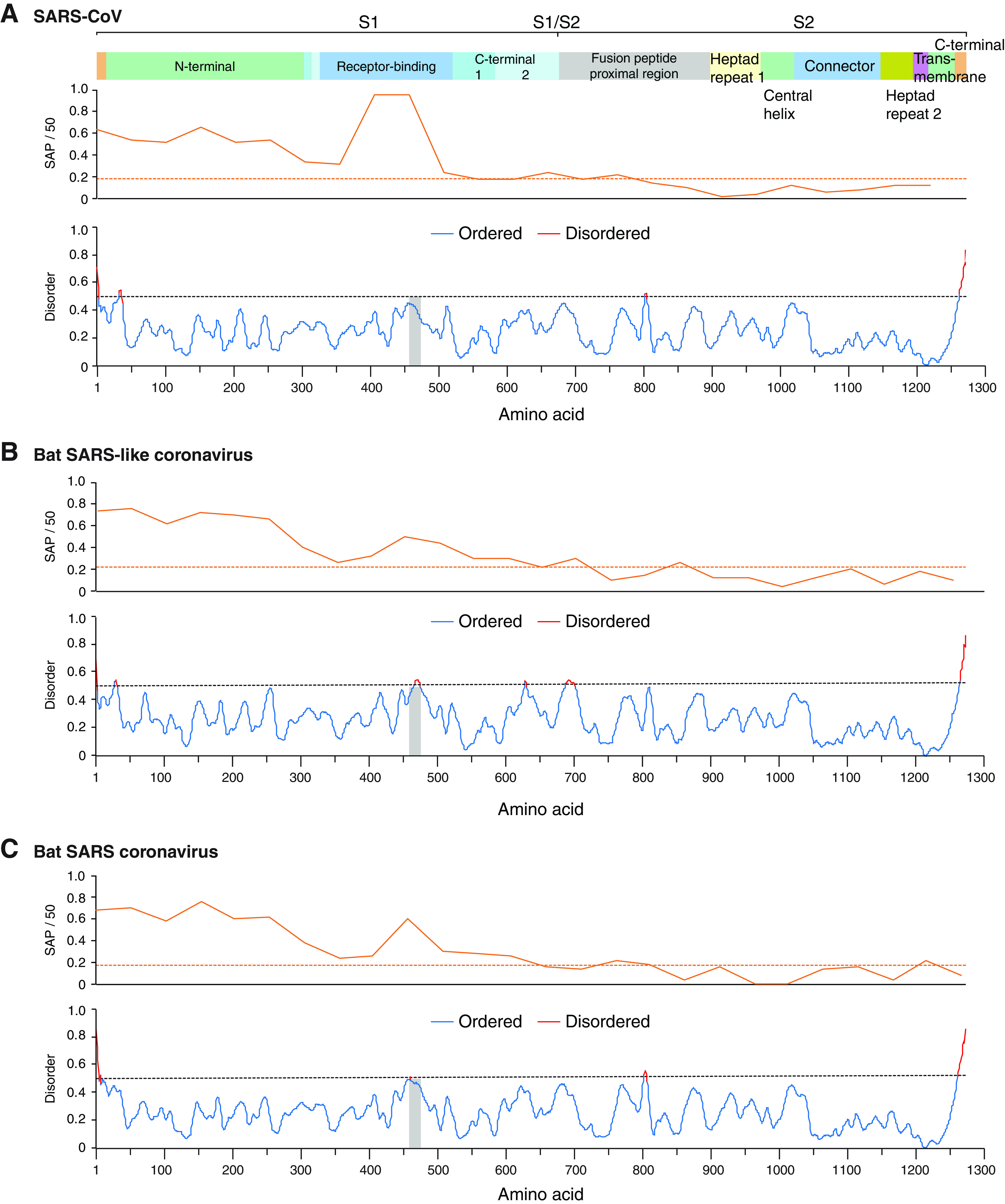
S protein amino acid variation and disorder in sarbecoviruses. Single-amino-acid polymorphism (SAP) and disorder/order of S protein. The average and 99% confidence interval are represented by the dotted line. The vertical gray line marks the location of the ACE2 receptor binding domain. (A) SARS-CoV-2. (B) Bat SARS-like coronavirus. (C) Bat SARS coronavirus.

Amino acid sequence in the S glycoprotein is variable in all species of the subgenus *Sarbecovirus* (Fig. 10). Variation localizes to subunit S1, particularly to the receptor binding domain, which is predicted to be intrinsically disordered for bat-SARS and bat-SARS-like coronaviruses (Fig. 10). Intrinsically disordered proteins mediate functional diversity and interactions with multiple partners (59, 60). These observations support the model that, in β-CoVs, the S glycoprotein is mutationally robust and contains disordered areas.

## DISCUSSION

Host and viral factors contribute to virus evolution (38, 61). The starting material is the introduction of mutations (nucleotide substitutions, insertions, or deletions) in the genome through RNA-dependent RNA polymerase errors during replication, RNA recombination, and reassortment (in segmented viruses) (39, 61). While they may occur randomly, selection separates beneficial from detrimental and neutral mutations. Selection is imposed by the host, the environment, and their interaction. Mutations that provide a beneficial advantage are more likely to be fixed in the genome (37). Under this scenario, the distributions of mutations in the viral genome are not random. Instead, mutations accumulate to higher than random frequencies in areas of the genome that contribute to fitness by enhancing stability, transmission, replication efficiency, escape from immunity, suppression of immunity responses, or a combination (37–39). The collective results of these effects may be evident through biological properties such as host adaptation, pathogenicity, or others.

The emergence of new species, such as SARS-CoV-2, and the rapid emergence of new SARS-CoV-2 strains are an indication that β-CoVs evolve quickly and have a high capacity to switch hosts and to adapt to new hosts. Results described here show that β-CoVs are more variable than polioviruses (49). In some species, variation is close to that observed for HIV-1 (Fig. 5 and 6). The wide host range observed across species suggests that, in β-CoVs, genomic variation is related to the genetic diversity of the host.

While mutations accumulate in the entire genome, they are not randomly or equally distributed. Instead, preferential accumulation of mutations in the S glycoprotein is a general feature of all members of the genus *Betacoronavirus* (Fig. 5B). This was particularly evident in SARS-CoV-2. Strains identified to date (16, 21) and isolates from early and middle 2020 can be distinguished based on the S glycoprotein sequence alone (Fig. 3).

In HIV-1, glycoproteins gp120 and gp41 are the most variable in the genome (Fig. 6). Both β-CoV S glycoprotein and HIV gp120 and gp41 are envelope proteins and mediate viral entry. The S glycoprotein binds to the ACE2 receptor (55), while gp120 binds to the CD4 receptor (62). Both the S glycoprotein and gp120 induced the formation of neutralizing antibodies. These features suggest several mechanisms driving diversifying selection in envelope glycoproteins: cellular receptors, entry cofactors, and antibodies.

Within the variety of coronavirus hosts, the cellular receptors, entry cofactors, and cellular proteases that process the S1/S2 cleavage site and immunity responses are likely diverse (12, 63). Results described here show that, for β-CoVs, the S glycoprotein is variable and mutationally robust and contains intrinsically disordered areas (Fig. 4, 5B, and 9). Disordered proteins allow functionality with a diverse set of interaction partners (44). These observations are consistent with a model in which host diversity pushes diversifying selection in the S glycoprotein. Mutational and structural robustness in the S glycoprotein provide a selection advantage, are major contributors to β-CoV evolution, and may lead to the emergence of new strains and species.

In the reference sequence Wuhan-Hu-1 (NC_045512.2), the S glycoprotein contains residues that are compatible, but not optimal, for binding human receptor ACE2 (55). Accordingly, SARS-CoV-2 has the potential to accumulate mutations for more efficient entry into human cells and to escape from neutralizing antibodies. Consistent with this model, SARS-CoV-2 strains detected to date mainly differ in the S glycoprotein (Fig. 1A) (16, 21). The D614G and Q677P mutations make the virus more transmissible and more pathogenic to humans (24, 45) and have been detected in several parts of the world (33, 64). Amino acids 614 and 677 are near hypervariable C-terminal domain 2 in subunit S1 (Fig. 4C). Furthermore, the D614G mutation and others in the receptor binding domain reduce affinity to monoclonal antibody CR3022 (64). In Mexico (65) and Wisconsin (Fig. 3), the H49Y mutation in the S protein was the most frequent and appears to have independent origins, suggesting convergent evolution.

In humans, the strength of the immune responses is not uniform, as indicated by immunocompromised patients (2, 20), and immunity-driven selection in SARS-CoV-2 has been documented (66). The human population is genetically diverse enough to select for variants in SARS-CoV and MERS-CoV (Fig. 9), and there is genetic variability in human leukocyte antigen genes that affect susceptibility to SARS-CoV-2 and the severity of the disease (67). Thus, it is likely that SARS-CoV-2 will continue to accumulate mutations for efficient transmission and genome replication and differentiate into biological strains as the virus faces selection pressure from genetically distinct human populations or immunocompromised individuals. For example, a 27-amino-acid deletion was detected in protein 7 in Arizona (68), new mutations formed sublineages in the southwest (24), and SARS-CoV-2 populations were grouped into clades in Mexico (65).

Our analysis identified proteins 3 and 7 and ORF8 as variable in SARS-CoV and bat SARS (Fig. 5C). The 3b protein is an inhibitor of the interferon response, and a variant with a longer 3b protein induces more severe symptoms and has enhanced ability to suppress induction of type I interferon (69). The 7a protein antagonizes antiviral factor BST-2 to enhance virion release (70). ORF8 is a cofactor of the RNA-dependent RNA polymerase and an inhibitor of the type I interferon response (71–73). Further, a 29-nt deletion in ORF8 attenuated SARS-CoV, and mutations or deletions in SARS-CoV-2 ORF8 caused attenuation. Although no difference was detected *in vivo*, higher replication was detected *in vitro* than in the wild-type virus (66, 74). ORF8 is different between SARS-CoV and SARS-CoV-2 and does not include functional motifs (50). Because ORF8 induces a robust antibody response, deletions may reflect immunity-driven selection (66). Due to their biological role in replication, suppression of antiviral defense, and hypervariable nature (Fig. 5C), proteins 3 and 7 and ORF8 are likely contributors to pathogenicity and host adaptation in sarbecoviruses.

Vaccines against SARS-CoV-2 induce neutralizing antibodies against the prefusion conformation of the S glycoprotein (8, 75, 76). However, nonneutralizing antibodies against subunit S2 are also developed (8). Variation within the S2 subunit is among the highest in the genome (Fig. 4). Nonneutralizing antibodies may provide a mechanism for the virus to escape from the immune response (77). Thus, variation in the S glycoprotein provides β-CoVs a mechanism to escape the immune response and an important selection advantage.

Vaccines and antiviral drugs might function as selection agents (36). In an infected individual, new variants are generated (26) and may be selected to escape neutralizing antibodies, which were developed against natural infection or triggered by a vaccine. Indeed, in immunocompromised patients, recurrent deletions in the N-terminal domain were detected, and these deletions mediate escape from neutralizing antibodies (20).

Factors contributing to β-CoV evolution include intrinsic properties of the S glycoprotein (mutationally robust and intrinsically disordered), natural genetic diversity in their hosts, and diversity in the strength of the immune response. Similarly, several factors contribute to SARS-CoV-2 differentiation into strains, including natural genetic diversity in the human population, diversity in the strength of the immune response, and, possibly, selection imposed by vaccines. This represents a challenge for vaccine development and deployment, because vaccines may only be efficient against closely related strains, ineffective against diverse strains, and fail to prevent reinfection.

## MATERIALS AND METHODS

All computational analyses were done on high-performance computing nodes. Custom scripts are available upon request.

### Genomic RNA sequences.

All available genomic sequences for the genus *Betacoronavirus* were obtained from NCBI (http://www.ncbi.nlm.nih.gov/) on 6 April 2020 using customized scripts based on Entrez Programming Utilities (E-utilities; https://eutils.ncbi.nlm.nih.gov/entrez/eutils/). For SARS-CoV-2, nucleotide accessions were redownloaded on 13 May 2020. For HIV-1, 100 random full-length sequences were obtained from NCBI to provide a representative sample of HIV-1 variation on 19 February 2021. Only accessions with at least 95% of the reference genome length were retained. For each species, the reference accession describing a complete genome was identified (Table 1).

### Genomic and amino acid variation.

For each species, nucleotide and amino acid variation analyses were conducted either on the entire genome or the spike S protein. Both were estimated in a 50-nt window. Nucleotide substitutions on the genome were measured based on nucleotide diversity (48) and genomic variation (40). Nucleotide diversity was calculated using TASSEL (https://www.maizegenetics.net/tassel) (78). Amino acid substitutions were measured based on SAPs (40). SNPs or SAPs were identified and mapped using SNP-sites version 2.4.1 (https://github.com/sanger-pathogens/snp-sites) (79) and VCFtools (80). The average and 99% confidence interval (*P* < 0.01) were estimated and plotted for each species. For variation per ORF, only ORFs present in at least 25% of the β-CoVs were counted.

### Annotated phylogram for the S protein.

Phylograms (40) were made using GraPhlAn (http://segatalab.cibio.unitn.it/tools/graphlan/) to illustrate the geographical location, host, and variation in the S protein (81).

### Disorder of the S protein.

Order/disorder was estimated using the Multilayered Fusion-based Disorder predictor (MFDp) with a false-positive rate of 5% (82). For each species, the amino acid sequence of the reference accession was used. Ordered and disordered areas are below and above the 0.5 threshold, respectively.

### Annotated phylogram of U.S. species.

All U.S. January sequences from the 23 March 2020 download were included in the early (January) time period. Three random sequences were chosen from each state with accessions from the late (July-August) time period to ensure a representative sample. Neighbor-joining phylogenetic trees were created using MAFFT version 7.4 (https://mafft.cbrc.jp/alignment/software/) with bootstrap values of 100. Accessions were aligned and mutations were identified using Geneious version 8.0 (https://www.geneious.com).

### Data availability.

All accession numbers used in this study were downloaded from GenBank (Table 1).
